# Supplementary material for: Ongoing movement controls sensory integration in the dorsolateral striatum
Source: Nat Commun. 2023 Feb 22;14:1004. doi: 10.1038/s41467-023-36648-0 (PMC9947004; doi:10.1038/s41467-023-36648-0)
Supplement: Supplementary file 1 — Supplementary Information [file 41467_2023_36648_MOESM1_ESM.pdf]

Supplementary material:

Ongoing movement controls sensory integration in the dorsolateral striatum

Roberto de la Torre-Martinez [1*], Maya Ketzef [1,2], Gilad Silberberg [1*,2].

[1] Department of Neuroscience, Karolinska Institutet, Stockholm 17177, Sweden.

[*]Correspondence: roberto.de.la.torre.martinez@ki.se & gilad.silberberg@ki.se

[2] These authors jointly supervised this work

Supplementary material in this file includes 6 figures and 2 tables.

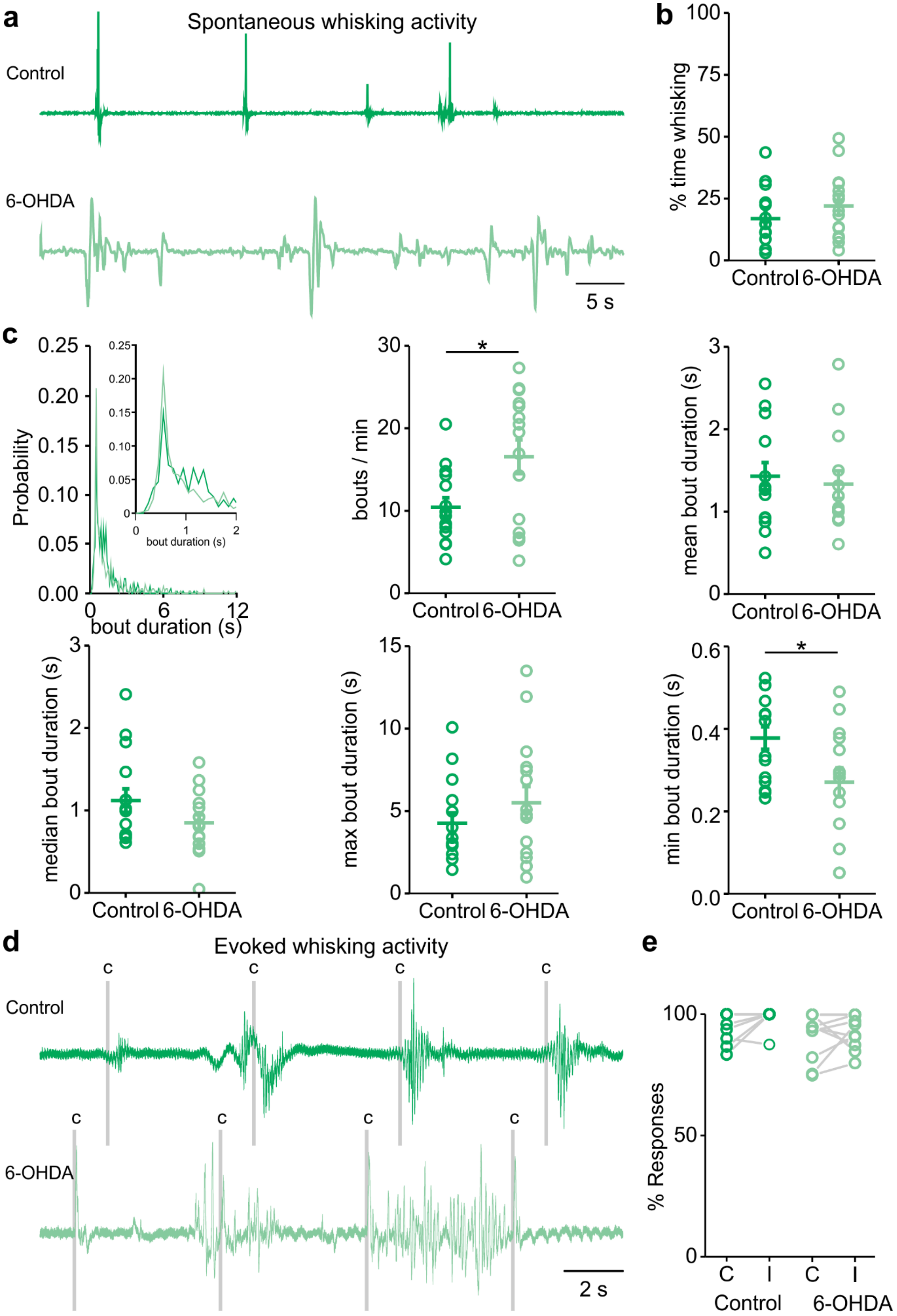

Supplementary Fig. 1

Supplementary Fig 1 (related to Fig. 2). Characteristics of spontaneous and evoked whisker activity in control and 6-OHDA lesioned mice.

a. Example traces representing spontaneous whisker activity of control (top, dark green) and 6-OHDA lesioned (bottom, light green) mice as detected by the infra-red beam. **b.** Fraction of the time the animal is performing active whisking. Control (n=15) and 6-OHDA lesioned mice (n=15). **c.** Whisking bouts duration and frequency in control and 6-OHDA lesioned mice. 6-OHDA lesioned mice demonstrated more whisking bouts per minute than control animals (top middle, control=10.43 ± 1.14 bouts/min, n=15 mice; 6-OHDA=16.56 ± 2.05 bouts/min, n=15 mice, two-sided unpaired t test, * $P = 0.01$) which were shorter in duration (bottom right, control=0.38 ± 0.03 s; 6-OHDA=0.27 ± 0.03 s, two-sided unpaired t test, * $P = 0.02$). Other parameters were not different between the groups. For (**b**) and (**c**) each circle represents the data from a single mouse and error bars indicate mean ± SEM. Unpaired t-test * $P < 0.05$. **d.** Example traces representing evoked whisker activity that could be delivered during spontaneous whisking or quiescence. Traces of control (top, green) and 6-OHDA lesioned (bottom, light green) mice are presented, and the trigger for air puff delivery is marked in gray. **e.** Percentage of ipsi- (I) and contralateral (C) air puff stimulations triggering whisking activity in control (n=16) and 6-OHDA lesioned mice (n=16). Each gray line represents the data from a single mouse and error bars indicate mean ± SEM. Paired t-test. Source data are provided as a Source Data file.

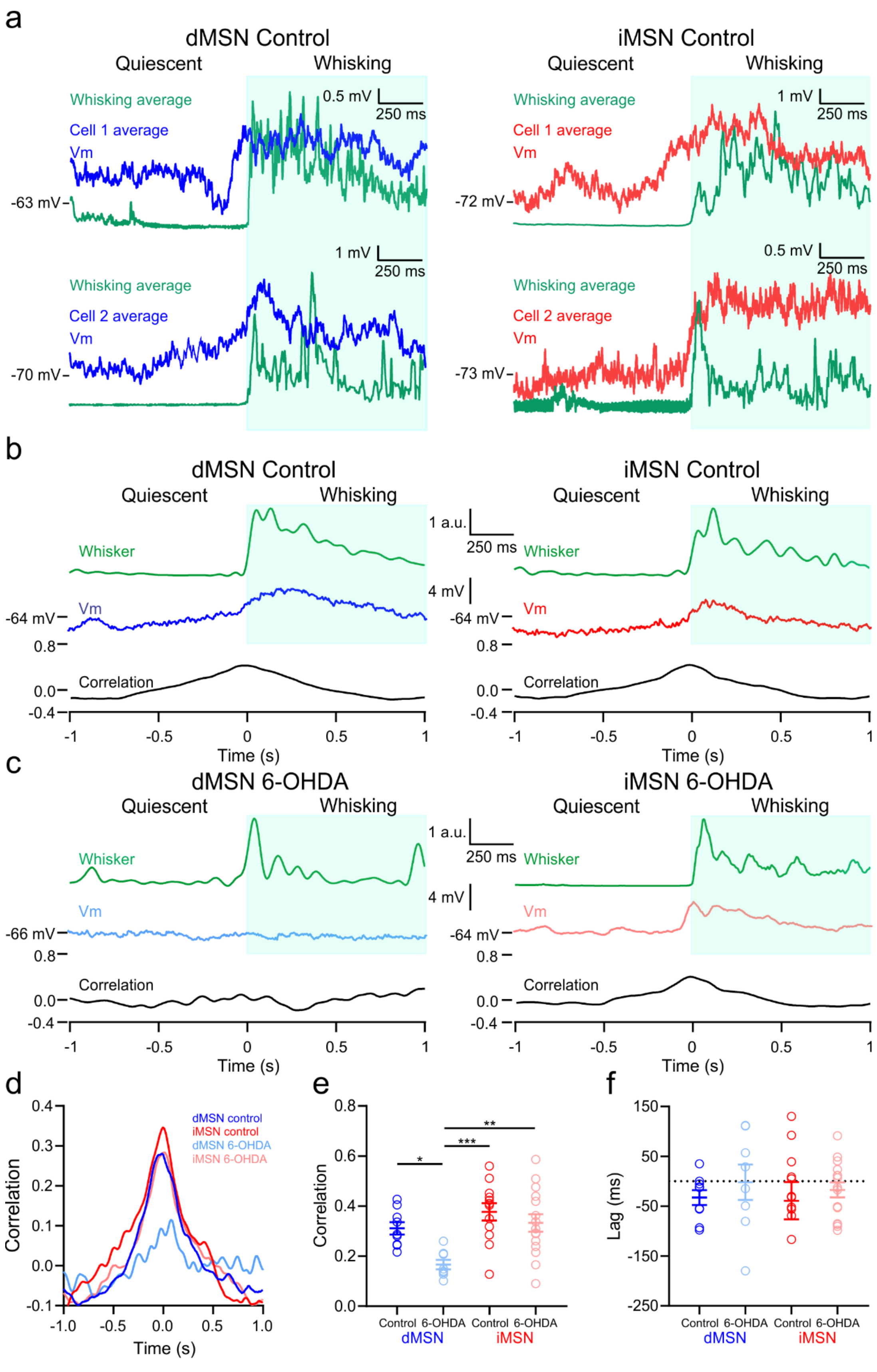

Supplementary Fig. 2

Supplementary Fig 2 (related to Fig. 3). Whisker activity is correlated with membrane depolarization of MSNs under control conditions, but not in dMSNs in the DA-depleted striatum.

a. Example of average whisker activity (green trace) and average membrane potential (Vm) from control dMSNs (blue traces, left) and control iMSNs (red traces, right) showing the transition from quiescence to whisking. **b.** Example of whisker activity transition from quiescence to whisking (green trace, top), average membrane potentials from a control dMSNs (blue trace, middle left) and a control iMSNs (red trace, middle right) and cross-correlation of the whisking envelope with the corresponding membrane potential, for a 2 second window which is 1 second before and 1 second after whisking onset (black trace, bottom panel). **c.** Same as in (**b**) but for 6-OHDA lesioned mice. **d.** Average cross-correlation obtained by averaging data from control dMSNs (n=9), control iMSNs (n=12), 6-OHDA dMSNs (n=8) and 6-OHDA iMSNs (n=15). **e.** Peak amplitude of the crosscorrelogram for the neurons represented in (**d**) (dMSN control=0.31 ± 0.025, n=9 cells; dMSN 6-OHDA=0.17 ± 0.019, n=8 cells, * p=0.043; dMSN 6-OHDA vs iMSN control= 0.38 ± 0.034, n=12 cells, *** $P < 0.001$, dMSN 6-OHDA vs iMSN 6-OHDA=0.33 ± 0.035, n=15 cells,

** $P = 0.006$; One-way ANOVA test). **f** Time lag of the peak of the crosscorrelogram for neurons represented in (**d**). For (**e**) and (**f**) each circle represents the data from a single neuron and error bars indicate mean ± SEM. Source data are provided as a Source Data file.

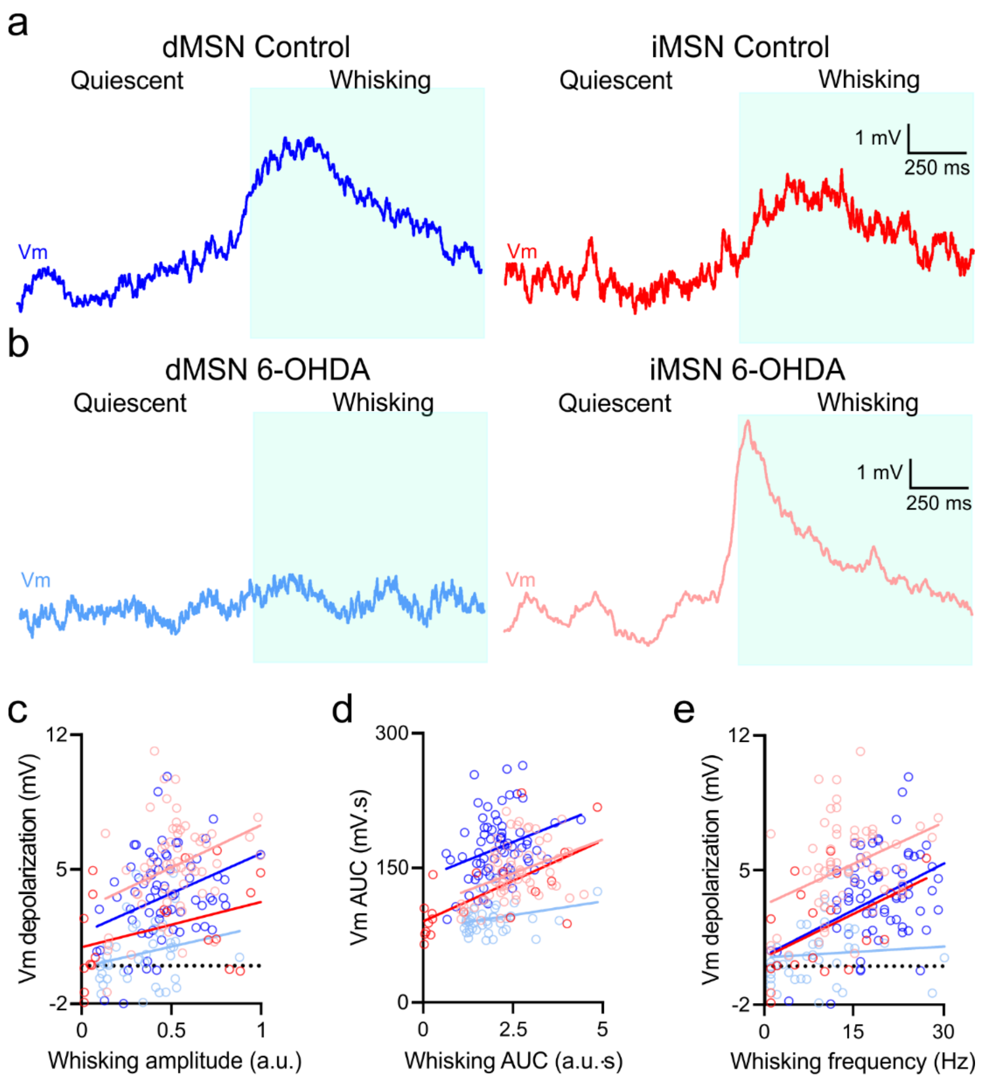

Supplementary Fig. 3

Supplementary Fig 3 (related to Fig. 3). Membrane depolarization of MSNs is correlated with whisking vigor under control conditions but not in dMSNs in the DA-depleted striatum.

a. Example of average membrane potential (Vm) depolarization of a dMSN (blue trace) and an iMSN (red trace) of control mice in the transition from quiescence to whisking. **b.** Same as in (**a**) for dMSN (light blue trace) and an iMSN (light red trace) of 6-OHDA lesioned mice. **c.** Distributions of the maximum membrane potential depolarization of the neurons showed in (**a**) and (**b**) in relation to the maximum amplitude of whisking. Each circle represents an individual whisking epoch (dMSN control 70 epochs, dMSN 6-OHDA 41 epochs, iMSN control 19 epochs, iMSN 6-OHDA 65 epochs). Solid lines present the slope (dMSN control, $r = 0.10$; $P = 0.006$, $F = 8.220$, iMSN control, $r = 0.13$; $P = 0.12$, $F =2.627$, 6-OHDA dMSN $r = 0.05$; $P = 0.14$, $F = 2.227$, 6-OHDA iMSN $r = 0.1125$; $P = 0.006$, $F = 7.984$, linear regression with F test). **d.** A plot of the area under the curve (AUC) for the membrane potential of the neurons showed in (**a**) and (**b**) in relation to the AUC of whisking 500 ms after whisking onset. Each circle represents an individual whisking epoch (same epochs as in **c**). Solid lines present the slope (dMSN control, $r = 0.09$; $P = 0.01$, $F =6.981$, iMSN control, $r = 0.41$; $P = 0.003$, $F = 11.76$, 6-OHDA dMSN $r = 0.07$; $P = 0.09$, $F =3.107$, 6-OHDA iMSN r = 0.21; $P < 0.001$, $F = 17.12$, linear regression with F test). **e.** Relationship of the maximum membrane potential depolarization and whisking frequency computed 500 ms after whisking onset. Each circle represents an individual whisking epoch (same epochs as in **c**). Solid lines present the slope (dMSN control, $r = 0.16$; $P < 0.001$, $F = 13.34$, iMSN control, $r = 0.23$; $P = 0.04$, $F = 4.989$, 6-OHDA dMSN $r = 0.01$; $P = 0.58$, $F =0.315$, 6-OHDA iMSN $r = 0.14$; $P = 0.002$, $F = 10.31$, linear regression with F test). Source data are provided as a Source Data file.

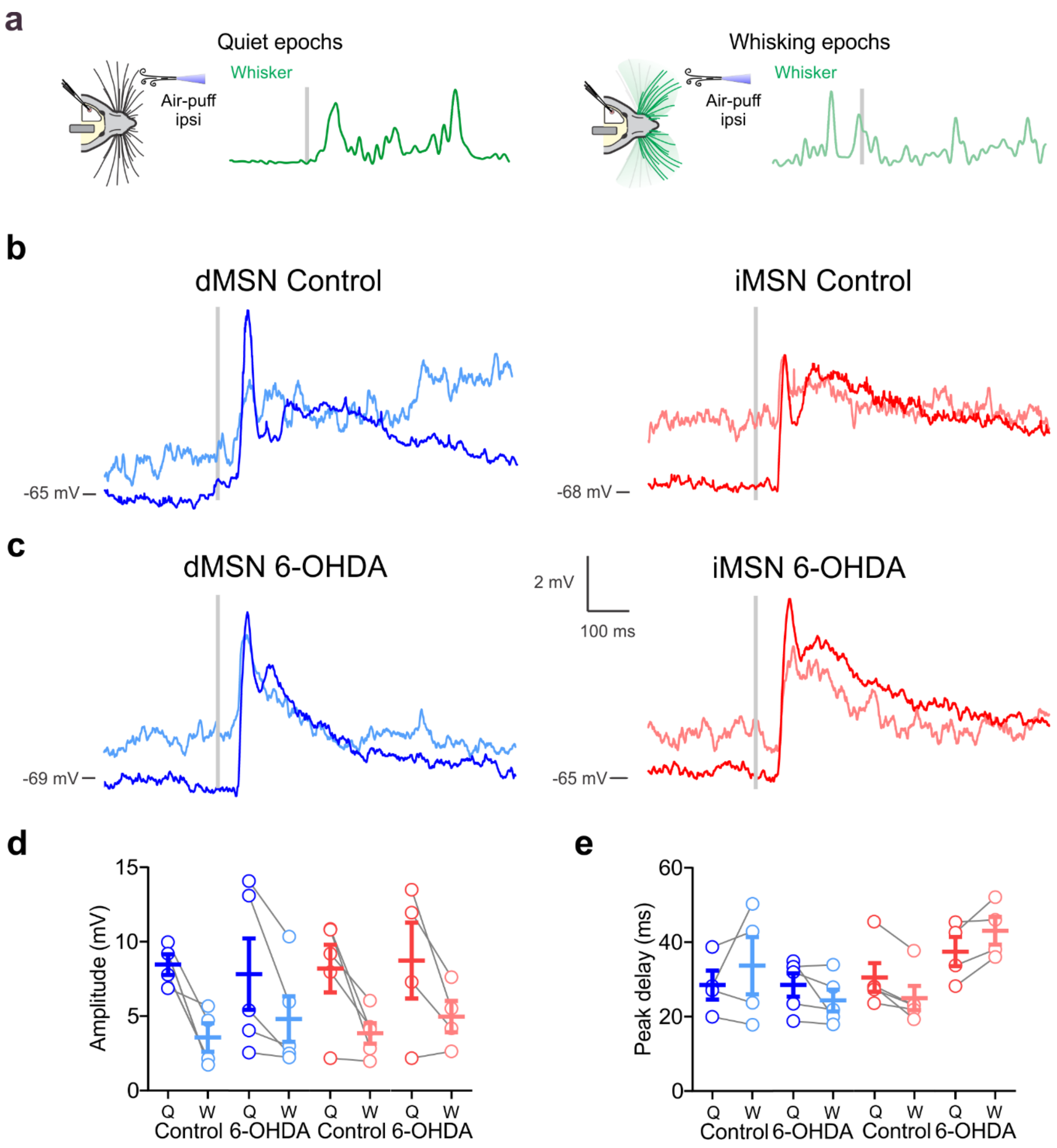

Supplementary Fig. 4

Supplementary Fig. 4 (related to Fig. 4). MSNs integrate ipsilateral tactile sensory information differently during quiescence and whisking in control and DA-depleted mice.

a. Schematic of ipsilateral whisker stimulation during quiet (Q) epochs (left) or during whisking (W) epochs (right). The stimulations were delivered randomly in intervals from 3 to 6 secs. Classification of events as occurring during Q or W was done post-hoc. The grey line indicates the trigger to stimulation. **b.** Grand average of traces from control dMSNs (blue, left, n=4 cells) and control iMSNs (red, right, n=5 cells) mice upon ipsilateral whisker stimulation (grey line). Dark color traces indicate the averaged response of MSNs to sensory stimulation during quiescence, while light color traces indicate responses to sensory stimulation during whisking in the same cell. Note the decrease in amplitude in the sensory responses evoked during whisker movement in both MSN types. **c.** Same as in (**b**) but for 6-OHDA lesioned mice. Dark color trace indicates MSNs responses to sensory stimulation during quiescence, while light color indicates responses to sensory stimulation during whisking. Grand average (n=5 cells in dMSNs and n=4 cells in iMSNs). **d-e.** Ipsilateral whisker deflections produce larger amplitude responses in both MSN types during quiescence in control and 6-OHDA lesioned mice (**d**) without affecting the peak of maximum depolarization (**e**, peak delay) (dMSNs control n=4 cells, iMSN control n=5 cells, dMSNs 6-OHDA n=5 cells, iMSN 6-OHDA n=4 cells). For (**d**) and (**e**), each grey line represents the data from a single cell during Q (dark color) and W (light color), and error bars indicate mean ± SEM. Source data are provided as a Source Data file.

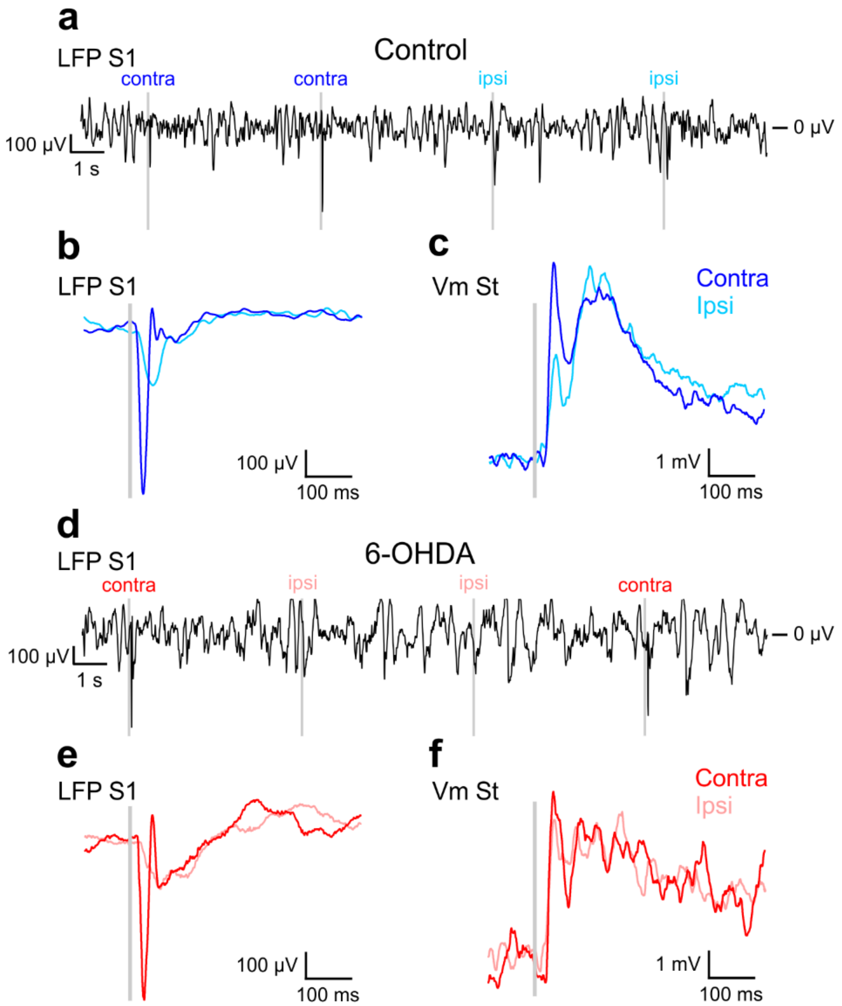

Supplementary Fig. 5

Supplementary Fig. 5 Laterality coding is impaired in MSNs but not in the somatosensory cortex of DA-depleted mice.

a. Example traces showing local field potential (LFP) activity in somatosensory cortex (S1) in control mice. The gray lines indicate the times of contralateral (contra, dark blue) and ipsilateral (ipsi, light blue) whisker stimulations. **b.** Average of LFP responses of somatosensory cortex in control mice to contralateral (dark blue traces) and ipsilateral (light blue traces) whisker stimulation. **c.** Average of responses of MSN in control mice to contralateral (dark blue traces) and ipsilateral (light blue traces) whisker stimulation. Both (**b**) and (**c**) were recorded in parallel and depict the average responses of cortex and striatum to the same whisker stimulations. **d.** Example traces showing LFP activity in somatosensory cortex in 6-OHDA lesioned mice. A gray line indicates the contralateral (contra, dark red) and ipsilateral (ipsi, light red) onset of the whisker stimulations. **e.** Average of LFP responses of somatosensory cortex in 6-OHDA lesioned mice to contralateral (dark red traces) and ipsilateral (light red traces) whisker stimulation. **f.** Average of responses of MSNs in 6-OHDA lesioned mice to contralateral (dark red traces) and ipsilateral (light red traces) whisker stimulation. Both (**e**) and (**f**) were recorded in parallel and depict the average responses of cortex and striatum to the same whisker stimulations. Source data are provided as a Source Data file.

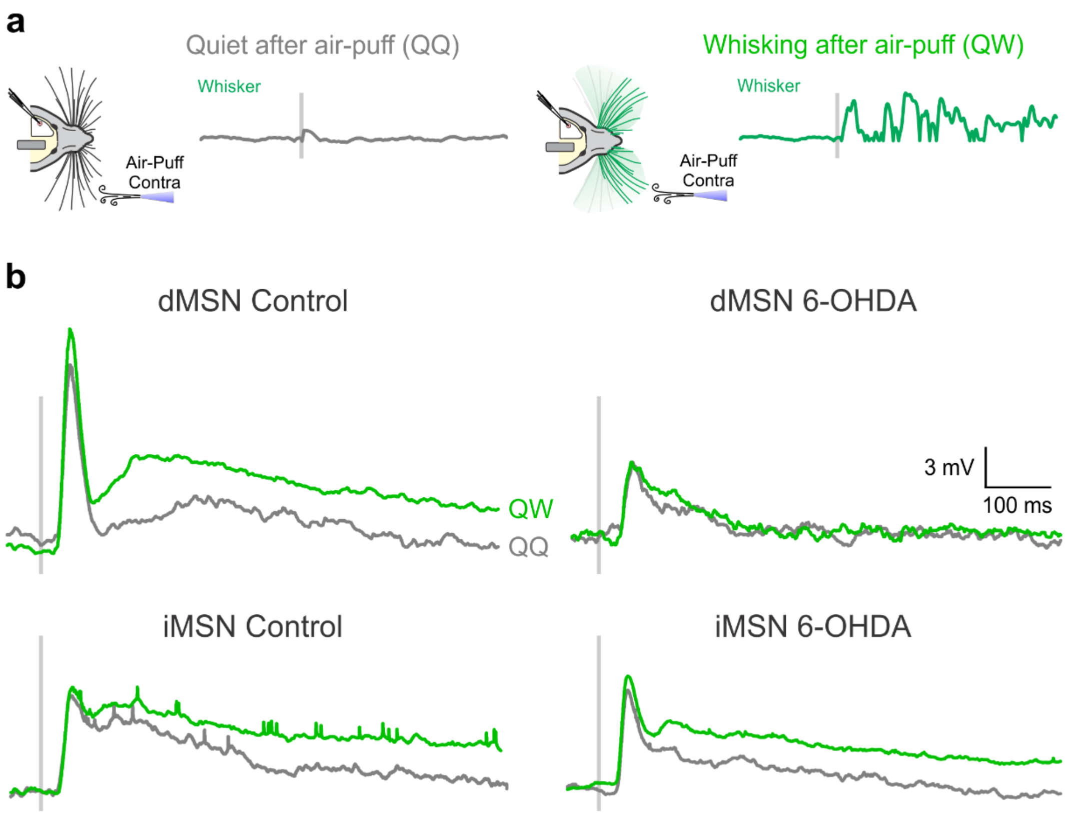

c

			Amplitude (mV)		Onset delay (ms)		AUC (mV^2)	
**Cell-type**	**Mice**	**Stimulus**	**Mean**	**SEM**	**Mean**	**SEM**	**Mean**	**SEM**
**dMSN**	Control	QW	5.93	2.07	87.72	12.21	11967.03	202.87
	Control	QQ	4.81	1.96	85.28	4.44	9967.11	2841.43
	6-OHDA	QW	0.50	-	82.22	-	2300.59	-
	6-OHDA	QQ	0.61	-	76.62	-	2500.66	-
**iMSN**	Control	QW	3.50	1.23	68.72	12.34	9740.35	3498.96
	Control	QQ	3.39	0.85	71.13	12.99	6600.39	1543.19
	6-OHDA	QW	1.95	0.43	75.16	10.93	6483.84	1490.65
	6-OHDA	QQ	1.76	0.39	75.98	9.06	4950.41	855.18

Supplementary Fig. 6

Supplementary Fig 6. The late component of the sensory response correlates with whisker movement and is reduced in dMSNs following DA depletion.

a. Schematic of contralateral whisker deflections during quiet epochs that do not promote whisker movement after the stimulation (QQ, left) and whisker deflections during quiet epochs that trigger whisker movement after the stimulation (QW, right). The light grey line indicates the moment the stimulation was delivered. **b.** Left: Grand average of membrane potential traces from dMSNs (top) and iMSNs (bottom) in control (left) and 6-OHDA (right) mice upon contralateral whisker deflections during QQ (grey trace) and whisker deflections during QW (green trace) (dMSN control n= 3, iMSN control n= 5, dMSN 6-OHDA n= 1, iMSN 6-OHDA n= 6). **c.** Late component of the tactile sensory response recorded in QQ and QW conditions in identified MSNs from control and 6-OHDA lesioned mice. Source data are provided as a Source Data file.

Supplementary Table 1 (related to Fig. 6): Short-latency responses to whisker stimulation in identified MSNs from control and DA-depleted mice.

			Amplitude (mV)		Onset delay (ms)		Peak delay (ms)		Slope (mV/ms)	
**Cell-type**	**Mice**	**Stimulus**	**Mean**	**SEM**	**Mean**	**SEM**	**Mean**	**SEM**	**Mean**	**SEM**
**dMSN**	control	Contra-	5.55	0.98	15.20	0.87	33.22	2.46	0.34	0.07
	control	Ipsi-	3.95	0.88	17.94	0.99	34.01	2.08	0.25	0.05
	6-OHDA	Contra-	6.27	1.22	12.54	0.89	33.45	3.31	0.35	0.06
	6-OHDA	Ipsi-	5.93	0.97	11.77	1.01	36.03	4.28	0.29	0.05
**iMSN**	control	Contra-	4.84	0.87	12.14	0.69	27.70	1.13	0.34	0.06
	control	Ipsi-	3.27	0.52	13.64	0.98	27.55	1.59	0.23	0.03
	6-OHDA	Contra-	7.26	1.03	13.74	0.71	33.70	1.96	0.43	0.05
	6-OHDA	Ipsi-	6.71	1.22	14.32	1.09	34.90	2.06	0.39	0.07

Responses to Contra- and Ipsilateral whisker stimulation, recorded in dMSNs and iMSNs. Sample size: dMSN control n= 13, iMSN control n= 13. dMSN 6-OHDA n=13, iMSN 6-OHDA n=13. Statistical significance for the different values is indicated in Fig. 6, and Results. Source data are provided as a Source Data file.

Supplementary Table 2 (related to Fig. 7): Late responses to whisker stimulation in identified MSNs from control and DA-depleted mice.

			Amplitude (mV)		Onset delay (ms)		AUC (mV^2)	
**Cell-type**	**Mice**	**Stimulus**	**Mean**	**SEM**	**Mean**	**SEM**	**Mean**	**SEM**
**dMSN**	control	Contra-	3.85	0.73	75.77	4.57	5651.54	559.53
	control	Ipsi-	4.01	0.87	72.87	5.65	6613.08	985.42
	6-OHDA	Contra-	2.06	0.45	67.83	6.15	5113.08	1190.29
	6-OHDA	Ipsi-	1.64	0.40	67.72	4.38	5767.69	1092.77
**iMSN**	control	Contra-	2.78	0.63	59.43	3.53	7803.33	1746.24
	control	Ipsi-	2.48	0.58	56.88	4.17	7212.27	1589.20
	6-OHDA	Contra-	2.79	0.36	65.21	1.50	7062.31	785.27
	6-OHDA	Ipsi-	2.26	0.39	73.14	7.95	7242.31	956.92

Responses to Contra-, Ipsi- whisker stimulation, recorded in dMSNs and iMSNs. Sample size: dMSN control n= 13, iMSN control n= 12. dMSN 6-OHDA n=13, iMSN 6-OHDA n=13. Statistical significance for the different values is indicated in Fig. 7, and Results. Source data are provided as a Source Data file.
