# Supplementary material for: Ongoing movement controls sensory integration in the dorsolateral striatum
Source: Nat Commun. 2023 Feb 22;14:1004. doi: 10.1038/s41467-023-36648-0 (PMC9947004; doi:10.1038/s41467-023-36648-0)
Supplement: Supplementary file 3 — Description of Additional Supplementary Files [file 41467_2023_36648_MOESM3_ESM.pdf]

File name: Supplementary Movie 1

Description: Contralateral whisker stimulation does not induce whisker movement in the ipsilateral whisker pad. A video showing air puff stimulation to the contralateral whiskers of an anesthetized mouse. Top panel: video showing the responses to air puffs delivered to ipsi- and contralateral whiskers, taken at 100 frames per second (FPS). The colored dots indicate the tracking of whiskers in DeepLabCut (DLC). Bottom panel: travelling one second time window (left) and fixed representation of the detected whisking movement (left) as analyzed by the software. The triggers for ipsilateral (red) and contralateral (blue) air puffs are indicated in black. Video is played at 30 FPS.

File name: Supplementary Movie 2

Description: Air puff stimulation induces whisking without aversive responses. A video showing air puff stimulation and responses of the whiskers and eye of the mouse. Top panel: video showing the mouse during delivery of air puff, taken at 100 frames per second (FPS). The colored dots indicate the tracking of the whisker and eye contour in DeepLabCut (DLC). Middle panel: eye size representing the area of the polygon formed by the four landmarks (blue, left) and whisking activity (red, right) as analyzed by DLC. Travelling one second time windows are shown. Bottom panel: parallel representation of the eye size and whisking activity with the trigger for the air puff (indicated in black). Video played at 30FPS.
