# Supplementary material for: Ongoing movement controls sensory integration in the dorsolateral striatum
Source: Nat Commun. 2023 Feb 22;14:1004. doi: 10.1038/s41467-023-36648-0 (PMC9947004; doi:10.1038/s41467-023-36648-0)
Supplement: Supplementary file 6 — Reporting Summary [file 41467_2023_36648_MOESM6_ESM.pdf]

nature portfolio

Corresponding author(s): Roberto de la Torre-Martinez, Gilad Silberberg

Last updated by author(s): Feb 8, 2023

Reporting Summary

Nature Portfolio wishes to improve the reproducibility of the work that we publish. This form provides structure for consistency and transparency in reporting. For further information on Nature Portfolio policies, see our Editorial Policies and the Editorial Policy Checklist.

Statistics

For all statistical analyses, confirm that the following items are present in the figure legend, table legend, main text, or Methods section.

n/a	Confirmed	
☐	☒	The exact sample size (*n*) for each experimental group/condition, given as a discrete number and unit of measurement
☐	☒	A statement on whether measurements were taken from distinct samples or whether the same sample was measured repeatedly
☐	☒	The statistical test(s) used AND whether they are one- or two-sided<br>*Only common tests should be described solely by name; describe more complex techniques in the Methods section.*
☐	☒	A description of all covariates tested
☐	☒	A description of any assumptions or corrections, such as tests of normality and adjustment for multiple comparisons
☐	☒	A full description of the statistical parameters including central tendency (e.g. means) or other basic estimates (e.g. regression coefficient) AND variation (e.g. standard deviation) or associated estimates of uncertainty (e.g. confidence intervals)
☐	☒	For null hypothesis testing, the test statistic (e.g. F, t, r) with confidence intervals, effect sizes, degrees of freedom and P value noted<br>*Give P values as exact values whenever suitable.*
☒	☐	For Bayesian analysis, information on the choice of priors and Markov chain Monte Carlo settings
☒	☐	For hierarchical and complex designs, identification of the appropriate level for tests and full reporting of outcomes
☐	☒	Estimates of effect sizes (e.g. Cohen's *d*, Pearson's *r*), indicating how they were calculated

Our web collection on statistics for biologists contains articles on many of the points above.

Software and code

Policy information about availability of computer code

Data collection	Local field potential recordings were acquired using a Differential AC Amplifier model 1700 (A-M Systems, USA) and digitized at 20 KHz with CED and Spike2 version 7 (Cambridge Electronic Design).<br>Whole-cell patch-clamp recordings acquired using MultiClamp 700B amplifier (Molecular Devices) and digitized at 20 KHz with a CED acquisition board and Spike2 software version 7 (Cambridge Electronic Design).<br>Whisker activity was acquired using an IR reflective sensor (HOA1405, 574 Honeywell, NC, USA) and digitized at 20 KHz with a CED acquisition board and Spike2 software version 7 (Cambridge Electronic Design).<br>For Supplementary Video1 and Video2, whisker activity and eye size was acquired at 100 FPS using a videocamera Grasshopper3 (GS3-U3-23S6M-C, FLIR Systems, Wilsonville) and FlyCapture 2.13.3.31 software (FLIR Systems, Wilsonville).
Data analysis	Electrophysiological data were analyzed using built-in functions in Matlab R2020a (Mathworks,USA).<br>Statistics were performed in Prism 8.0 (GraphPad Software, USA).<br>Supplementary Video1 and Video2 whisker activity and eye size was analyzed using standard built-in functions in DeepLabCut 2.2.

For manuscripts utilizing custom algorithms or software that are central to the research but not yet described in published literature, software must be made available to editors and reviewers. We strongly encourage code deposition in a community repository (e.g. GitHub). See the Nature Portfolio guidelines for submitting code & software for further information.

Data

Policy information about availability of data

All manuscripts must include a data availability statement. This statement should provide the following information, where applicable:

- Accession codes, unique identifiers, or web links for publicly available datasets
- A description of any restrictions on data availability
- For clinical datasets or third party data, please ensure that the statement adheres to our policy

A source data file supporting the findings of this study is provided with this paper and its supplementary information files. Further information and requests for resources and reagents should be directed to and will be fulfilled by the corresponding author. Source data are provided with this paper.

Analysis was done using standard built-in functions in Prism 8, Matlab and DeepLabCut.

Human research participants

Policy information about studies involving human research participants and Sex and Gender in Research.

Reporting on sex and gender	N/A
Population characteristics	N/A
Recruitment	N/A
Ethics oversight	N/A

Note that full information on the approval of the study protocol must also be provided in the manuscript.

Field-specific reporting

Please select the one below that is the best fit for your research. If you are not sure, read the appropriate sections before making your selection.

☒ Life sciences ☐ Behavioural & social sciences ☐ Ecological, evolutionary & environmental sciences

For a reference copy of the document with all sections, see nature.com/documents/nr-reporting-summary-flat.pdf

Life sciences study design

All studies must disclose on these points even when the disclosure is negative.

Sample size	No sample size calculations were performed. Blind recording in deep structures mean catching cells by chance, thus it is impossible to predict when a cell from a small population will be recorded from. In agreement with previous publications, we used similar cell numbers (E.g. DOI: 10.1016/j.neuron.2017.05.004, DOI: 10.1016/j.celrep.2016.05.026, DOI: 10.1016/j.cub.2020.11.019)
Data exclusions	We excluded neurons with abnormal resting potential, indicating a bad patch configuration.<br>To ensure the robustness of our finding, we pre-established that experiments with less than 10 whisking epochs were not considered to calculate the mean membrane potential and variance of the neurons.<br>To avoid ambiguous situations between quiscence and whisking and ensure the robustness of our finding, we pre-stablish that whisking epochs shorter than 500 ms were not considered. This condition did not apply to the analysis in Supplementary Fig1, where we explicitly analyze the length of all whisking epochs.<br>To ensure the robustness of our finding, we pre-established that experiments with less than 10 sensory stimulations were discarded for the analysis of sensory responses.
Replication	Replication was done accross trials, recorded cells and animals. The exact number of repetitions (cells or animals) are indicated in the figures or figure legends. In addition, the data displayed enables the appreciation of the population results on top of the mean value.
Randomization	Mice from both sexes, ages between 2 and 3 months old, were selected on availability at the time of the initial procedure. Allocation of animals into experimental groups was not randomized. All animals were treated identically.<br>Randomized 15 ms air puff stimulations (ipsi- and contralateral) were delivered at a range of 0.2 to 0.33 Hz.
Blinding	Blinding was not possible since the mouse lines were always a known factor. The correct localization of the recorded cells and expression of channelrhodopsin was examined post hoc.

Reporting for specific materials, systems and methods

We require information from authors about some types of materials, experimental systems and methods used in many studies. Here, indicate whether each material, system or method listed is relevant to your study. If you are not sure if a list item applies to your research, read the appropriate section before selecting a response.

Materials & experimental systems

n/a	Involved in the study
☐	☒ Antibodies
☒	☐ Eukaryotic cell lines
☒	☐ Palaeontology and archaeology
☐	☒ Animals and other organisms
☒	☐ Clinical data
☒	☐ Dual use research of concern

Methods

n/a	Involved in the study
☒	☐ ChIP-seq
☒	☐ Flow cytometry
☒	☐ MRI-based neuroimaging

Antibodies

Antibodies used	Cy3 conjugated streptavidin diluted 1:1000 (Jackson ImmunoResearch Laboratories, Cat: 016-160-084)<br>Rabbit anti-TH polyclonal antibody (Millipore, Cat: AB152) diluted 1:1000<br>Cy3-conjugated goat anti-rabbit polyclonal secondary antibody (Jackson Laboratories, Cat: 111-165-003) diluted 1:500
Validation	The antibodies were used as described in previous published works (for ex. https://doi.org/10.1016/j.neuron.2017.05.004 or https//doi.org/10.1523/JNEUROSCI.0852-07.2007)

Animals and other research organisms

Policy information about studies involving animals; ARRIVE guidelines recommended for reporting animal research, and Sex and Gender in Research

Laboratory animals	All experiments were performed according to the guidelines of the Stockholm municipal committee for animal experiments under an ethical permit to G.S. (N12/15). Mice of both sexes between 2 and 3 months of age were housed in groups from two to five in polycarbonate individually ventilated cages equipped with environmental enrichment (carton tubes and wood sticks). The cages were kept under constant temperature and humidity with a 12h light/dark cycle and ad libitum access to food and water<br><br>Mouse:B6.FVB(Cg)-Tg(Drd1-cre)EY217Gsat/Mmucd, GENSAT, MMRRC_034258-UCD<br>Mouse: STOCK Tg(Drd2-cre)ER44Gsat/Mmucd, GENSAT, MMRRC_017263-UCD<br>Mouse: STOCK Tg(Adora2a-cre)KG139Gsat/Mmucd, GENSAT, MMRRC_031168-UCD<br>Mouse: B6;129S-Gt(ROSA)26Sortm32(CAG-COP4*H134R/EYFP)Hze/J, the Jackson Laboratory, IMSR_JAX:012569
Wild animals	This study did not involve wild animals
Reporting on sex	Both male and female mice were used in the study, following a previous study (10.1016/j.neuron.2014.07.033) in which no differences were observed between sexes
Field-collected samples	This study did not involve samples collected from the field
Ethics oversight	All experiments were performed according to the guidelines of the Stockholm municipal committee for animal experiments under an ethical permits to G.S. (N2022/2020).

Note that full information on the approval of the study protocol must also be provided in the manuscript.
